# The Function, Role and Process of DDX58 in Heart Failure and Human Cancers

**DOI:** 10.3389/fonc.2022.911309

**Published:** 2022-06-22

**Authors:** Ping Yu, Peng Liang, Shifeng Pang, Wenjian Yuan, Yuxiang Zhao, Qiaojuan Huang

**Affiliations:** ^1^ Department of Cardiology, The Second Affiliated Hospital of Guangxi Medical University, Nanning, China; ^2^ United New Drug Research and Development Center, Biotrans Technology Co., LTD., Ningbo, China; ^3^ Institute of Bioengineering, Biotrans Technology Co., LTD., Ningbo, China

**Keywords:** heart failure, cancer, immune infiltration, DDX58, ischemic cardiomyopathy

## Abstract

**Background:**

Heart failure (HF) is the most common outcome of cardiovascular disease, and an increasing number of patients with heart failure die from noncardiac causes, such as cancer. Epidemiological data suggest that ischemic cardiomyopathy–induced HF (ischemic HF) may be associated with an increased incidence of cancer. This study aimed to investigate the possible mechanisms of the association between ischemic HF and cancer, as well as potential therapeutic targets.

**Methods:**

Weighted gene co-expression network analysis was performed to analyze the correlations between phenotypes and gene modules using immune cells as phenotypes. Differential analysis was then performed to screen differentially expressed genes (DEGs) in ischemic HF and normal control samples. The macrophage-related Brown module was identified as the key module, and immune-related DEGs were obtained by taking the intersection of the Brown module, DEGs, and immune-related genes using a Venn diagram. DDX58 was identified as the key gene using a protein–protein interaction network and expression analyses and validated using immunohistochemistry. Kaplan–Meier survival analysis was performed to analyze the correlation between DDX58 expression and tumor prognosis. Spearman correlation analysis was performed to assess the correlation between DDX58 expression and immune cell infiltration.

**Results:**

DDX58 was identified as a key immune-related gene associated with ischemic HF and was highly expressed in most cancer types. The survival analysis revealed a significant negative correlation between high DDX58 expression and prognosis in multiple tumor types. Moreover, DDX58 expression was significantly associated with immune cell infiltration and immune checkpoint gene expression in many cancer types.

**Conclusion:**

DDX58 is a key immune-related gene in ischemic HF and may play a crucial role in the relationship between ischemic HF and cancer. Pan-cancer analysis suggests that DDX58 is a promising clinical prognostic marker for most cancers and may be a therapeutic target for cancer patients and ischemic HF patients at an increased risk of cancer.

## Introduction

Heart failure (HF) is a complex and severe syndrome occurring when the heart is unable to deliver sufficient blood to meet its own needs under normal filling pressures (1). HF currently affects more than 60 million people worldwide, and the number continues to increase (2). The major causes of HF include coronary artery disease, hypertension, diabetes, and obesity, and the risk increases over time (3). The phenotype of HF patients varies across regions. In Africa, the main causes are hypertensive heart disease and dilated cardiomyopathy. In Asia and South America, the main cause is coronary artery disease. In Western countries, ischemic causes are significantly more prominent than in East Asia (4–6).

HF treatment techniques have improved in recent years. However, an increasing number of HF patients die from noncardiac causes, such as cancer (7, 8). Growing evidence suggests a causal relationship between HF and cancer (8). In addition to the increased risk of HF due to cancer itself and its treatments (9–11), patients with ischemic cardiomyopathy–induced HF (ischemic HF) have a 71% higher risk of developing cancer. This trend usually emerges 1.5 years after HF diagnosis and is most pronounced in HF patients with reduced ejection fractions (7, 12, 13). Epidemiological data suggest that HF patients diagnosed with cancer are more prone to in-hospital complications and death (14), and the European Society of Cardiology has called on medical professionals to pay more attention to the incidence of cancer in HF patients (15). However, due to the complexity of the interaction between HF and cancer, the pathophysiological mechanisms involved are unclear.

Immune system dysfunction appears to play a crucial role in myocardial remodeling and cancer development after myocardial infarction (16, 17). The monocyte system may be an important link between HF and cancer (18). The advent of immunotherapy has ushered in a new direction in the treatment of cancer and other autoimmune diseases, such as cardiovascular disease. In clinical practice, immunotherapies targeting various immune checkpoints have shown significant efficacy against various cancer types. However, currently available immune checkpoint inhibitors are expensive and have many cardiotoxic side effects, the most common of which is myocarditis (19). Myocarditis induced by immunodetection site inhibitors has been reported to have morbidity and mortality rates of up to 50% (20). Therefore, the discovery of safer and more effective therapeutic approaches is of great importance for the treatment of cancer and other autoimmune diseases. In addition, the recently proposed therapeutic strategy for improving DDX58-mediated innate immunity can be used as a complementary mechanism to compensate for the lack of immune checkpoint therapy (21, 22). To that end, this study aimed to explore the role of ischemic HF immune-related genes in pan-cancer through a comprehensive bioinformatics analysis, hoping to gain new insights into the association between ischemic HF and cancer and to reveal potential therapeutic targets that can help reduce the incidence of cancer in ischemic HF patients.

## Materials and Methods

### Data Sources

Twelve cases of ischemic HF and five normal control samples were downloaded from the GSE42955 dataset of the Gene Expression Omnibus (GEO) database, and 19 cases of HF and five normal control samples were downloaded from the GSE26887 dataset. Expression data for various tumor cell lines were downloaded from the Cancer Cell Line Encyclopedia (CCLE) database (23). Gene expression profiles of 33 cancers and normal tissues were obtained from The Cancer Genome Atlas (TCGA) and Genotype-Tissue Expression (GTEx) databases (24). A total of 7398 immune-related genes were downloaded from InnateDB (25).

### Single-Sample Gene Set Enrichment Analysis

The Immune Cell Abundance Identifier (ImmuCellAI) tool (http://bioinfo.life.hust.edu.cn/ImmuCellAI#!/ ) was used to estimate GSE42955 microarray data for single-sample Gene Set Enrichment Analysis (ssGSEA) to observe the infiltration abundance of 24 immune cells in ischemic HF and normal control samples, including 18 T cell subtypes and six other immune cells: DC, B cells, monocytes, macrophages, NK, neutrophils, CD4^+^ T, CD8^+^ T, NKT, Tgd, CD4 naive, CD8 naive, Tc, Tex, Tr1, nTreg, iTreg, Th1, Th2, Th17, Tfh, Tcm, Tem, and MAIT. Pearson correlation analysis was performed to assess the correlations between gene expression and immune cell infiltration. A value of *P* < 0.05 was considered statistically significant.

### Weighted Gene Co-Expression Network Analysis

Weighted gene co-expression network analysis (WGCNA) was performed using the R package “WGCNA” to identify gene modules associated with key immune cells in GSE42955. A soft threshold of β = 14 was chosen to achieve a scale-free topology by generating an adjacency matrix between pairs of genes using Pearson correlation analysis. Gene dendrograms and module colors were created using phase dissimilarity. The most significant gene modules (26) were identified by assessing the correlations between phenotypes and module genes.

### Analysis of Variance

The samples in the GSE42955 dataset were divided into ischemic HF and normal control samples, and differential analysis was performed using the R package “limma.” The results were expressed as volcano plots with a screening threshold of |log2FC| > 0.3785 for differentially expressed genes (DEGs) at *P* < 0.05. Venn diagrams were used to take intersections of significant DEGs, key WGCNA modules, and immune-related genes to obtain immune-related DEGs (IRDEGs).

### Protein–Protein Interaction Network

Metascape (https://metascape.org/gp/index.html#/main/step1) was used to analyze the biological functions of IRDEGs (27). Additionally, the obtained IRDEGs were imported into the STRING database (https://string-db.org/) to obtain a network of interactions. The top 10 most important genes were identified using the MCC algorithm in the CytoHubba plugin in Cytoscape software.

### Expression of Key Genes

The samples were divided into two parts based on the clinical characteristics or median expression of macrophages to compare the expression levels of key genes in different groups. Violin plots were used to represent the expression levels of genes or immune cells. Sankey plots were used to visualize the distribution of samples in different subgroups.

### Immunohistochemical Validation

Myocardial tissues from rats with HF after myocardial infarction and rats in a sham-operated group were used for immunohistochemical analysis. Animal experiment ethics has been approved by The Animal Care & Welfare Committee of Guangxi Medical University (No.201904027), and all experimental operations ensure animal ethical norms are followed. Briefly, after antigen repair and endogenous peroxidase elimination, sections were placed in a goat serum blocking solution at 37°C for 10–15 min. They were then incubated overnight at 4°C with a primary antibody against DDX58 (1:100; Affinity). The sections were subsequently rinsed with PBS three times and then incubated with a secondary antibody at 37°C for 20 min to develop color and hematoxylin lines for restaining. Samples were observed and imaged using light microscopy (Olympus, Tokyo, Japan).

### Functional and Pathway Clustering

DEG enrichment analysis was performed to identify significant Gene Ontology (GO) terms and Kyoto Encyclopedia of Genes and Genomes (KEGG) pathways. The level of statistical significance was set to *P* < 0.05. Moreover, ssGSEA was used to identify the KEGG and Hallmark pathways of the samples (28). Thresholds of |NES| > 1, NOM *P* < 0.05, and FDR q < 0.25 were used.

### Expression and Survival Analyses

Kaplan–Meier survival analysis was performed to assess the expression levels of DDX58 in different tumor tissues and analyze the correlations between DDX58 expression and overall survival (OS), disease-specific survival (DSS), and progression-free interval (PFI) in cancer patients. Univariate Cox regression analysis was performed to determine the hazard ratio (HR), with HR > 1 indicating high DDX58 expression as a high-risk factor, and a log-rank test was used to determine the *P*-values, with the level of statistical significance set to *P* < 0.05.

### Immune Infiltration Analysis

The correlation between DDX58 expression levels and immune cell infiltration was analyzed in 33 cancers using the Tumor Immune Evaluation Resource (TIMER) database and the CIBERSORT algorithm. Spearman correlation analysis was performed to determine the *P*-values (29). The immune, stromal, and ESTIMATE scores of different tumors were calculated using the R package “ESTIMATE” to predict tumor purity (30, 31) and to assess the correlations between the scores and DDX58 expression. Furthermore, 47 common immune checkpoint genes were collected, and their correlations with DDX58 expression were assessed using Spearman rank correlation analysis. Values of *P* < 0.05 were considered statistically significant.

## Results

### Immune Infiltration in Ischemic HF

**Figure 1:**
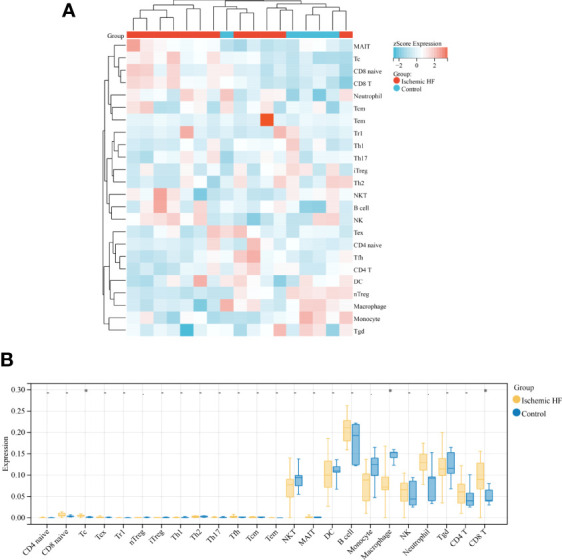
Comparison of immune infiltration in HF and normal control samples **(A)** Heat map of immune infiltration between Ischemic HF and Control samples based on ssGSEA score analysis; **(B)** Comparison of immune cell infiltration in Ischemic HF and Control samples. *P < 0.05.

In recent years, the importance of immune cells has been demonstrated in various diseases, including HF (32, 33). In this study, immune cell infiltration into ischemic HF and normal control tissues was first analyzed using ssGSEA. A heat map was constructed to visualize the infiltration of 24 immune cells into HF and normal control tissues (**Figure 1A**). The infiltration abundances of these immune cells were then compared. The results showed that the abundance of Tc and CD8^+^ T cells was significantly higher in HF than in normal control samples, whereas that of macrophages was significantly lower in HF samples (**Figure 1B**). These results suggest that the inflammatory response generated by these immune cells may be associated with ischemic HF.

### Genetic Features Associated With Immune Cells

**Figure 2:**
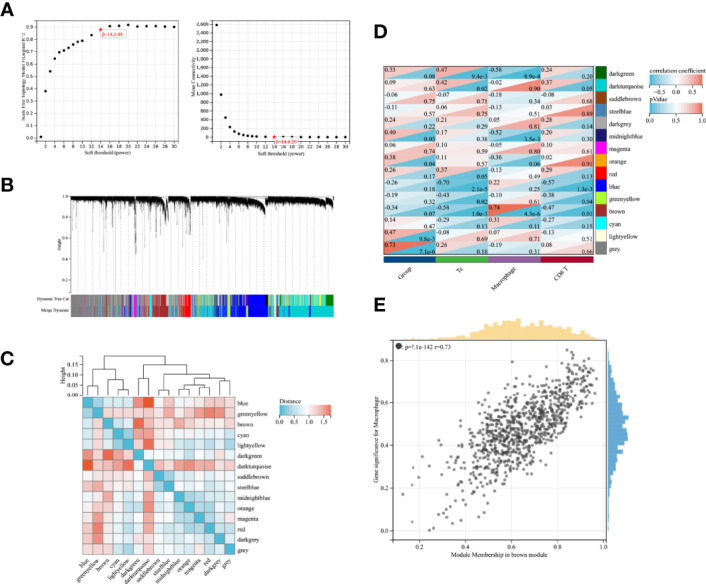
WGCNA screening of gene modules associated with immune infiltration **(A)** Scale-free fit indices and mean connectivity for soft thresholds; **(B)** Dendrogram of immune-associated genes based on phase dissimilarity metric clustering; **(C)** Module feature vector clustering; **(D)** Heat map of gene modules with feature correlation; **(E)** Module feature genes associated with Macrophage abundance in brown modules.

WGCNA was performed to further investigate the genetic features associated with immune cell infiltration. **Figure 2A** shows the scale-free fit indices and average connectivity for various soft threshold capabilities. The genes were divided into 15 modules using hierarchical clustering analysis and represented using branches of the clustering tree and different colors (**Figures 2B, C**). Heat maps were created by calculating the correlations between the gene models and phenotypes (**Figure 2D**). All gene modules showed weak correlations with Tc and CD8^+^ T cells, with correlation values less than 0.5. The Brown module had the strongest correlation with macrophages (r = 0.73, *P* = 7.1e-142; **Figure 2E**).

### Analysis of Variance

**Figure 3:**
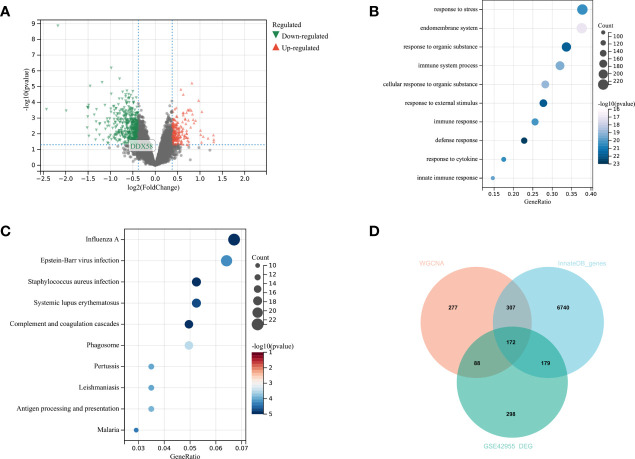
Analysis of differences in GSE42955 **(A)** Volcano plot of significant DEGs between ischemic heart failure and normal control samples; **(B, C)** top 10 enriched GO terms and KEGG pathways of DEGs, respectively; **(D)** Venn diagram of DEGs, WGCNA key modules and immune gene sets.

A total of 737 DEGs, including 547 downregulated and 190 upregulated genes, were identified in GSE42955 and compared with normal control samples (**Figure 3A**). The biological functions of DEGs were subsequently investigated using GO and KEGG pathway enrichment analyses. Most DEGs were enriched in GO terms related to immune responses, such as immune system processes and innate immune responses (**Figure 3B**). Most of the enriched KEGG pathways were associated with diseases such as influenza A, systemic lupus erythematosus (SLE), and pertussis, as well as with immune-related pathways such as complement and coagulation cascade reactions, antigen processing, and presentation (**Figure 3C**). DEG, in the Brown module gene, and InnateDB immune-related gene overlapping to obtain 172 immune-related DEGs (**Figure 3D**).

### Selection of Key Genes

**Figure 4:**
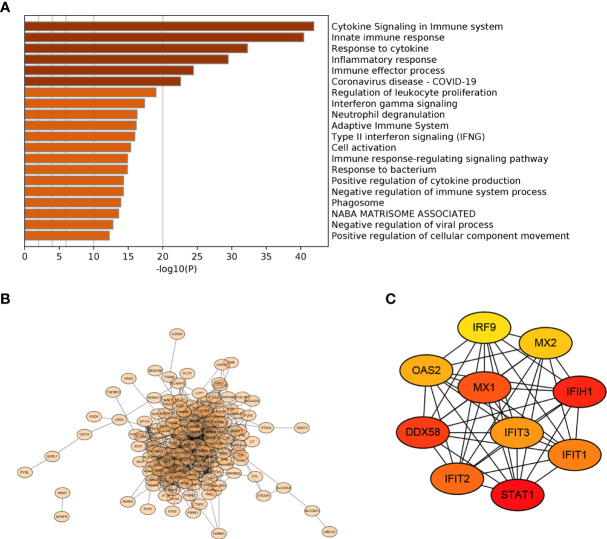
PPI selection of key genes **(A)** Functional terms of 172 IRDEGs; **(B)** PPI network diagram of 172 IRDEGs; **(C)** Top 10 important genes in the MCC algorithm.

Functional enrichment analysis showed that 172 IRDEGs were mainly involved in the signaling and regulation of cytokines in the immune system, regulation of the immune system and immune response, interferon signaling, and response to and regulation of bacteria or viruses (**Figure 4A**). A network diagram was drawn by analyzing the functional role relationships between these IRDEGs (**Figure 4B**). Ten genes were obtained using the MCC algorithm: IRF9, MX1, MX2, IFIH1, IFIT1, IFIT2, IFIT3, STAT1, DDX58, and OAS2 (**Figure 4C**).

### Validation of Potential Ischemic HF Biomarkers

**Figure 5:**
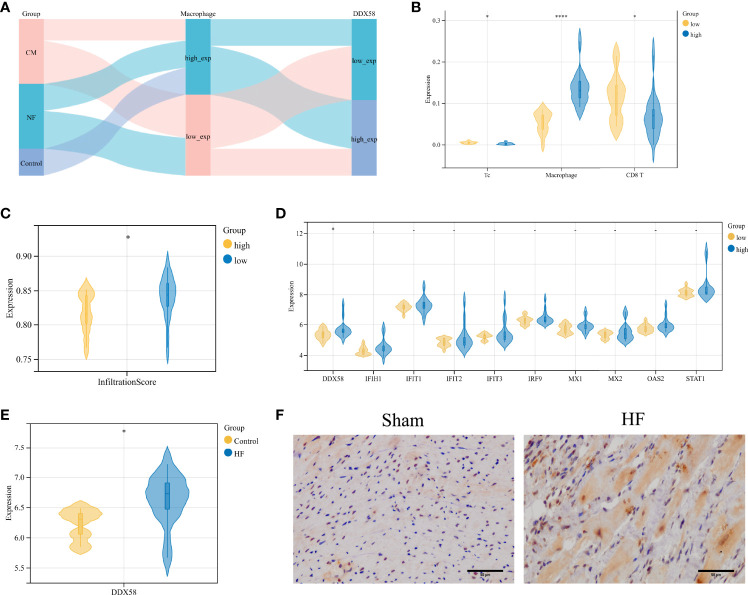
Validation of potential biomarkers for ischemic heart failure **(A)** Sankey diagram of GSE42955; **(B)** Comparison of Macrophage, Tc, CD8^+^ T cell infiltration abundance in high and low Macrophage infiltration groups in GSE42955; **(C)** Differences in immune infiltration abundance in high Macrophage infiltration and low Macrophage infiltration groups; **(D)** Differences in expression levels of 10 genes in high Macrophage infiltration and low Macrophage infiltration abundance; **(E)** Expression level of DDX58 in GSE26887; **(F)** IHC staining of DDX58 in post-infarction heart failure group and sham-operated group (×400). *P < 0.05, ****P < 0.0001.

The Sankey diagram in **Figure 5A** shows the distribution among sample types, macrophage infiltration abundance, and high and low DDX58 expression in GSE42955. In the high macrophage infiltration group, Tc cells showed lower infiltration levels, while macrophages and CD8^+^ T cells showed higher infiltration abundances (**Figure 5B**). The infiltration levels of immune cells were also lower in the high macrophage infiltration group (**Figure 5C**). Among the 10 key genes, only DDX58 was differentially expressed in this group (**Figure 5D**). High DDX58 expression was also observed in the HF samples (**Figure 5E**). These results suggest that high DDX58 levels are closely associated with both high macrophage infiltration levels and HF. Immunohistochemistry was performed to verify the expression levels of DDX58 in rat HF samples. The results showed significantly higher DDX58 expression in the post-infarction HF samples than in the sham-operated group (**Figure 5F**).

### Biological Functions of DDX58

**Figure 6:**
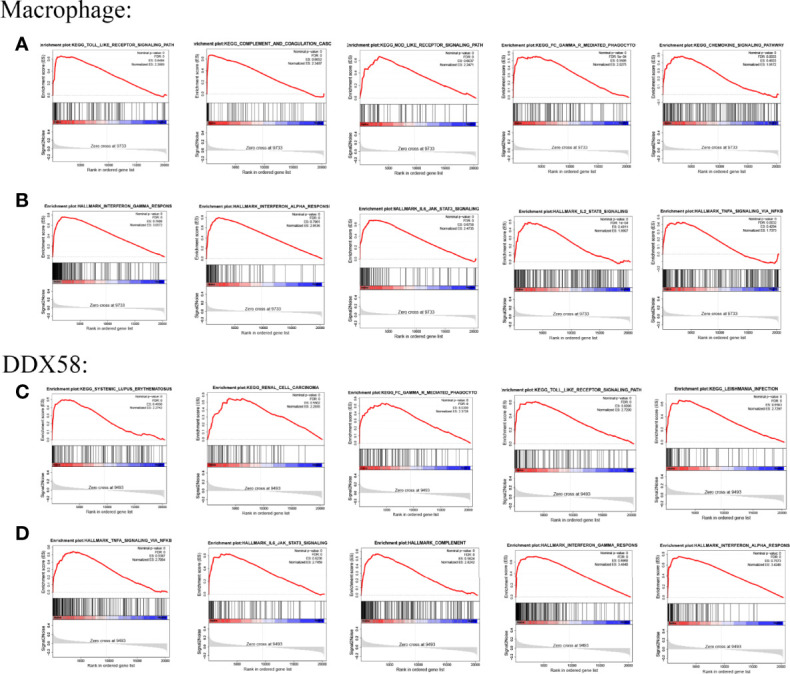
GSEA results of Macrophage and DDX58 **(A, B)** are the KEGG pathway and Hallmark pathway of Macrophage, respectively; **(C, D)** are the KEGG pathway and Hallmark pathway of DDX58, respectively.

GSEA was performed to evaluate the potential biological mechanisms of macrophages. The main KEGG pathways associated with the immune response were the TOLL-like receptor signaling pathway, complement and coagulation cascades, NOD-like receptor signaling pathway, FCγR-mediated phagocytosis, and chemokine signaling pathway (**Figure 6A**). The main Hallmark pathways were interferon γ response, interferon α response, IL6 JAK STAT3 signaling, IL2 STAT5 signaling, and TNFA signaling *via* NFKB (**Figure 6B**). The biological function of DDX58 in HF was then evaluated. Significant enrichment was observed in immune response and inflammation-related diseases such as SLE and FCγR-mediated phagocytosis and the TOLL-like receptor signaling pathway and other KEGG pathways (**Figure 6C**), TNFA signaling *via* NFKB, IL6 JAK STAT3 signaling, interferon γ response, interferon α response, and other Hallmark pathways (**Figure 6D**). Thus, the pathways enriched by macrophages and DDX58 were similar.

### Expression of DDX58 in Different Cancer Types

**Figure 7:**
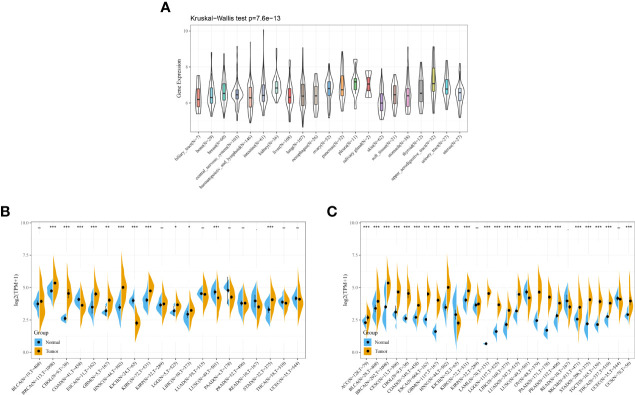
Expression of DDX58 in different cancer types **(A)** Expression level of DDX58 in different cancer cell lines; **(B)** Expression level of DDX58 in TCGA; **(C)** Expression level of DDX58 in the combination of GTEx database and TCGA. *P < 0.05, **P < 0.01, ***P < 0.001.

The expression of DDX58 in various cancer types was analyzed to explore its relationship with pan-cancer. An analysis based on the CCLE database showed that DDX58 was stably expressed in 21 tumor cell lines (**Figure 7A**). An analysis based on the TCGA database identified higher DDX58 expression in Breast invasive carcinoma (BRCA), Cholangiocarcinoma (CHOL), Esophageal carcinoma (ESCA), Glioblastoma multiforme (GBM), Head and Neck squamous cell carcinoma (HNSC), Kidney renal clear cell carcinoma (KIRC), Brain Lower Grade Glioma (LGG), Liver hepatocellular carcinoma (LIHC), and Stomach adenocarcinoma (STAD) than in normal tissues (**Figure 7B**). To compensate for the lack of normal tissues, a combined analysis using the GTEx and TCGA databases showed that DDX58 was highly expressed in most tumor types (**Figure 7C**). These findings suggest that DDX58 expression is closely related to tumorigenesis.

### Prognostic Potential of DDX58 in Pan-Cancer

**Figure 8:**
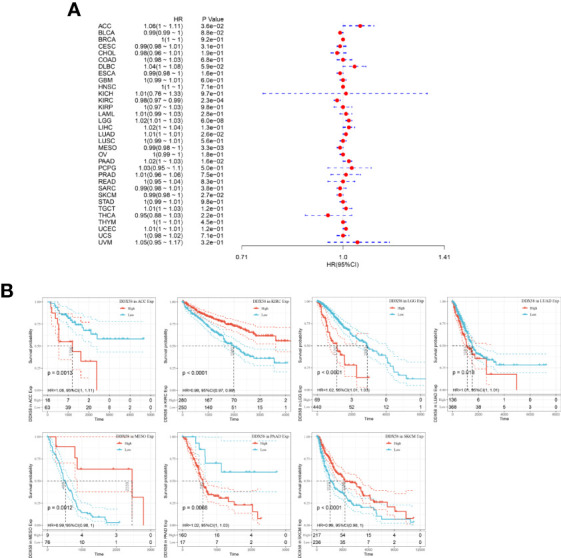
Relationship between DDX58 expression levels and overall survival of patients **(A)** Forest plot of the risk ratio of DDX58 in human pan-cancer; **(B)** Kaplan-Meier OS curves of DDX58 in the seven most significantly associated tumors.

Survival analysis is a common method for disease prognosis studies and is often used to explore the impact of prognostic factors on disease outcomes. The analysis of the correlation between DDX58 expression and OS in patients with cancer revealed a significant negative correlation between DDX58 expression and OS in Adrenocortical carcinoma (ACC), LGG, Lung adenocarcinoma (LUAD), and Pancreatic adenocarcinoma (PAAD) and a significant positive correlation in KIRC, Mesothelioma (MESO), and skin cutaneous melanoma (SKCM) (**Figure 8**). The DSS analysis showed that DDX58 expression negatively correlated with DSS in Lymphoid Neoplasm Diffuse Large B-cell Lymphoma (DLBC), LGG, LUAD, PAAD, and Uterine Corpus Endometrial Carcinoma (UCEC) and positively correlated with DSS in KIRC and SKCM (**Supplementary Figure 1**). The PFI analysis showed that high DDX58 expression significantly attenuated PFI in patients with six tumors: DLBC, LGG, LUAD, PAAD, Prostate adenocarcinoma (PRAD), and UCEC (**Supplementary Figure 2**). These findings indicate that DDX58 expression affects the prognosis of various tumors, suggesting that DDX58 may be a pan-cancer prognostic indicator.

### Correlation Between DDX58 and Cancer Immune Infiltration

**Figure 9:**
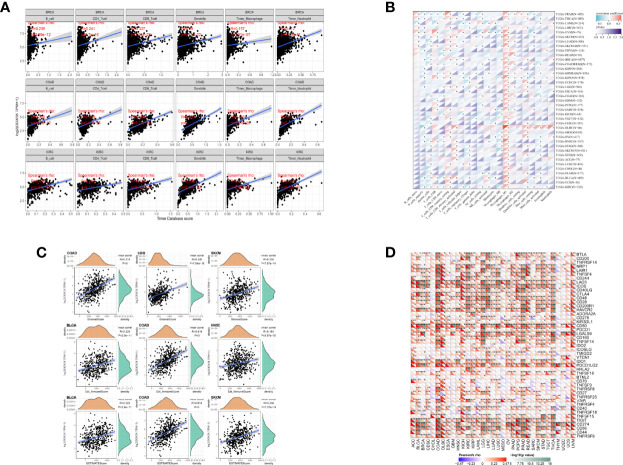
Correlation between DDX58 expression levels and cancer immune infiltration **(A)** Relationship between DDX58 expression levels and immune infiltration in the three most significantly associated tumors; **(B)** Heat map of the relationship between 22 immune cells and DDX58 in 33 tumors using CIBERSORT analysis; **(C)** Relationship between DDX58 expression levels and immune score, stromal score and ESTIMATE score in the three most significantly associated tumor types, respectively. **(D)** Correlation of DDX58 expression levels with immune checkpoint gene expression. *P < 0.05, **P < 0.01, ***P < 0.001.

Immune cell infiltration plays an important role in the progression and prognostic links of tumors. The analysis of the correlation between DDX58 expression and immune cell infiltration in different tumor tissues showed that DDX58 expression significantly correlated with the abundance of six types of immune cell infiltration in most cancers, including BRCA, Colon adenocarcinoma (COAD), KIRC, LGG, LIHC, Lung squamous cell carcinoma (LUSC), Ovarian serous cystadenocarcinoma (OV), PAAD, PRAD, Rectum adenocarcinoma (READ), and SKCM. A significant correlation was not observed only in Uveal Melanoma (UVM). In the immune cell infiltration analysis of 33 cancer types, DDX58 expression significantly correlated with macrophages in 24 types (**Figure 9A**). In the analysis of the correlations between DDX58 expression and 22 immune cells in 33 tumors using CIBERSORT, Macrophage_M1 showed the most prominent expression (**Figure 9B**). The analysis of the correlations between DDX58 expression and the immune, stromal, and ESTIMATE scores revealed significant correlations with all three scores in 20 cancer types. The three cancer types most strongly correlating with the immune score were Bladder Urothelial Carcinoma (BLCA), COAD, and HNSC. The three cancer types most strongly correlating with the stromal score were COAD, LGG, and SKCM. The three cancer types most strongly correlating with the ESTIMATE score were BLCA, COAD, and SKCM (**Figure 9C**). This suggests that DDX58 may be involved in the regulation of tumor immune cell infiltration. To examine whether DDX58 could be a target for tumor immunotherapy, the correlations between its expression levels and immune checkpoint genes were investigated. The results showed that DDX58 expression significantly correlated with the expression of 47 immune checkpoint genes in most cancer types, 44 in COAD, and 43 in Thyroid carcinoma (THCA) (**Figure 9D**). Overall, these results suggest that DDX58 plays an important role in tumor immune infiltration.

## Discussion

HF caused by ischemic heart disease is a common cause of morbidity and mortality in developed countries. Although pharmacological treatments and interventions have improved the OS of HF patients over the past 20 years, the prevalence of HF and hospitalization rates continue to increase (34). Moreover, although the development of therapeutic techniques such as early thrombolysis or coronary revascularization has greatly reduced HF-associated mortality, mortality with noncardiac causes, such as cancer, has increased. A significant proportion of HF patients develop cancer (7, 8), which has drawn attention to the biological HF–cancer intersection mechanisms. Cardiac oncology mostly focuses on the study of cancer therapy–related cardiac dysfunction (CTRCD). However, besides CTRCD, epidemiological and experimental studies suggest that HF may be a cancer susceptibility disease, leading to a new direction in cardiac oncology called reverse cardio-oncology (7, 12, 13, 18, 35). Recent evidence suggests that immune cell reprogramming may be a mechanism of HF-induced tumor growth. However, the associated pathophysiological mechanisms have yet to be elucidated (18). Therefore, in this study, we analyzed ischemic HF for immune cell infiltration and identified DDX58 as an important biomarker using comprehensive bioinformatics. We also investigated DDX58 expression, prognosis, and immune cell infiltration in various cancer types through pan-cancer analysis, confirming that DDX58 is a potential therapeutic target for ischemic HF patients at an increased risk of developing cancer.

Our enrichment analysis of DEGs between ischemic HF and normal control samples suggests that they are significantly associated with biological processes or pathways related to the immune system or innate immune responses. The innate immune system includes immune cells and cytokines, which mediate the onset and development of inflammation. The innate immune responses are triggered by receptors widely expressed on the surfaces of immune cells or in cytoplasmic lysates (36). Previous studies have shown that immune cells such as macrophages, mast cells, monocytes, neutrophils, B cells, and T cells reside in or infiltrate cardiac tissue (37). We found significant differences in the expression of Tc, CD8^+^ T cells, and macrophages between ischemic HF and normal tissue samples. T lymphocyte–mediated immune responses play an important role in HF. CD8^+^ T cells systematically expand in chronic ischemic HF, and their depletion is independently associated with death (38–40). Moreover, in the cardiovascular system, macrophages maintain homeostasis by removing senescent cells and promoting angiogenesis (41).

Our WGCNA suggests that only the gene modules associated with macrophages are related to ischemic HF. Macrophages play an important role in tissue inflammation and wound healing and are thought to be a major contributor to the inflammatory and fibrotic processes in HF (42, 43). They release CC chemokines in myocardial infarction to recruit large numbers of monocytes and induce differentiation into macrophages for phagocytosis, a process that can be mediated by many proteins (44). In line with Wei et al. (45), we screened 10 central genes using protein–protein interaction network analysis and identified the only immune-related gene associated with ischemic HF: DDX58. DExD/H-box helicase 58 (DDX58, also known as RIG-I) is a protein involved in viral double-stranded RNA recognition and type I IFN production and was originally described as a key mediator of antiviral and innate immune responses (46). It has been reported that autophagy mediates the degradation of DDX58 (47). Autophagy is a biological process that employs phagocytosis to prevent pathogen invasion in host cells. Cells such as macrophages and neutrophils are specialized phagocytes (48). Mature phagocytes can be converted into autophagosomes that transport cargo to lysosomes for degradation (49). Autophagy can affect antiviral immune responses by selectively degrading downregulated immune factors—for example, by disrupting the interaction between DDX58 and LRRC25, which leads to the degradation of DDX58, thereby stabilizing the DDX58 protein to positively regulate DDX58-mediated type I IFN signaling (47, 50). Moreover, the RIG-I-like receptor signaling pathway is closely associated with monocyte infiltration, and its activation may be associated with inflammation and cardiomyocyte apoptosis (51, 52). However, there is no direct evidence of a direct relationship between DDX58 and the development of ischemic HF. Our study is the first to identify significantly high DDX58 expression in ischemic HF tissues and a high degree of macrophage infiltration.

Our pan-cancer analysis indicates significantly higher DDX58 expression in most cancer types than in normal tissues. Our survival analysis suggests that high DDX58 expression is significantly associated with poor OS, DSS, and PFI in three cancer types: LGG, LUAD, and PAAD. This suggests that DDX58 may act as an oncogene with varying prognostic significance in different cancer types and that DDX58-targeting therapy may provide prognostic benefits to cancer patients. Immune cell reprogramming may be a mechanism of HF-induced tumor growth (18). Our correlation analyses suggest that DDX58 expression is significantly associated with immune cell infiltration in various cancer types, with Macrophage_M1 being the most prominent, indicating that the monocyte/macrophage system may be a key mediator of DDX58 and immune cell infiltration in the tumor microenvironment. Evidence suggests that monocytes/macrophages are involved in tumor growth, metastasis, and tumor vascularization by regulating the tumor microenvironment and that they play a key role in accelerating breast cancer growth after myocardial infarction (18, 53). It can thus be concluded that the centralized regulation of the innate immune system in ischemic HF exerts multiple tumorigenic effects that may lead to myocardial carcinogenesis.

Our correlation analyses also suggest that DDX58 expression significantly correlates with the expression of 47 immune checkpoint genes in most cancer types. Although immunotherapy targeting various immune checkpoints has shown significant efficacy in some cancer types, it is effective only against some “hot” tumors with heavy T cell infiltration. Moreover, the high cardiotoxicity of immune checkpoint inhibitors limits their use. For these reasons, a novel therapeutic approach to enhancing tumor immunogenicity has recently been proposed, namely the activation of RIG-I-mediated innate immunity in the tumor microenvironment (21, 54). It has also been reported that the RIG-I-mediated innate immune response may serve as a complementary mechanism for enhancing the anticancer efficacy of immune checkpoint therapies (55). All these pieces of evidence suggest that DDX58 is closely related to tumor immunotherapy.

In conclusion, this study shows that DDX58 is an important immune-related gene significantly associated with immune cell infiltration and tumor prognosis. Considering the role of DDX58 in ischemic HF and cancer, it can be hypothesized that the innate immune response is a potential mechanism of ischemic HF–induced tumor growth, in which DDX58-mediated monocyte/macrophage infiltration plays a crucial role. Therefore, immunotherapy targeting DDX58 may reduce the incidence of cancer in ischemic HF patients. Although the comprehensive analysis of DDX58 in this study is based on different databases and algorithms, there are still some limitations. First of all, DDX58 has not been reported in Ischemic HF, and this study only verifies the expression relationship between DDX58 and Ischemic HF, and the potential mechanism of DDX58 in Ischemic HF should be explored and verified in subsequent studies. Secondly, the role of DDX58 in cancer in this study is based on microarray data and bioinformatics analysis, and further *in vivo* or *in vitro* models should be established to elucidate the potential mechanism of DDX58 in Ischemic HF-induced tumor growth, and whether the innate immune response is a key link. Finally, prospective studies should be conducted in patients with Associated HF with cancer to evaluate the efficacy of DDX58 in reducing the cancer rate in patients with Ischemic HF.

In 2012, the European Society of Medical Oncology first published authoritative guidelines for the field of cardio-oncology (56), but in fact it was not until 2016 that the European Society of Cardiology and the American Society of Clinical Oncology issued two guidelines that the field of cardio-oncology had a real programmatic guideline (57, 58). To date, several countries around the world have actively set up independent diagnosis and treatment units of cardio-oncology, but cardio-oncology as a new and interdisciplinary discipline still faces serious challenges. Due to the lack of scientific and effective multidisciplinary diagnosis and treatment mechanism and related research data on cardio-oncology, clinicians face certain risks in the diagnosis and treatment process. The proposal of reverse cardio-oncology offers a whole new direction in cardiac oncology, and the active research on the potential pathophysiological mechanisms between heart failure and cancer and related epidemiological investigations will enrich the knowledge system of cardio-oncology and provide a theoretical basis and effective targets for the treatment of cardiac oncology diseases.

## Data Availability Statement

The datasets presented in this study can be found in online repositories. The names of the repository/repositories and accession number(s) can be found in the article/**Supplementary Material**.

## Ethics Statement

The animal study was reviewed and approved by The Animal Care and Welfare Committee of Guangxi Medical University.

## Author Contributions

All authors participated in the analysis process and wrote manuscripts of relevant sections. All authors have read and approved the publication of this study before submission.

## Conflict of Interest

YZ is employed by Biotrans Technology Co., LTD.

The remaining authors declare that the research was conducted in the absence of any commercial or financial relationships that could be construed as a potential conflict of interest.

## Publisher’s Note

All claims expressed in this article are solely those of the authors and do not necessarily represent those of their affiliated organizations, or those of the publisher, the editors and the reviewers. Any product that may be evaluated in this article, or claim that may be made by its manufacturer, is not guaranteed or endorsed by the publisher.

## Supplementary Material

Supplementary Figure 1 Relationship between DDX58 expression levels and disease-specific survival (DSS) **(A)** Forest plot of the risk ratio of DDX58 in human pan-cancer; **(B)** Kaplan-Meier DSS curves of DDX58 in the seven most significantly associated tumors.

Supplementary Figure 2 Relationship between DDX58 expression levels and progression-free interval (PFI). **(A)** Forest plot of the risk ratio of DDX58 in human pan-cancer; **(B)** Kaplan-Meier PFI curves of DDX58 in the seven most significantly associated tumors.
