# Supplementary figures and images for: The Function, Role and Process of DDX58 in Heart Failure and Human Cancers
Source: Front Oncol. 2022 Jun 22;12:911309. doi: 10.3389/fonc.2022.911309 (PMC9257035; doi:10.3389/fonc.2022.911309)

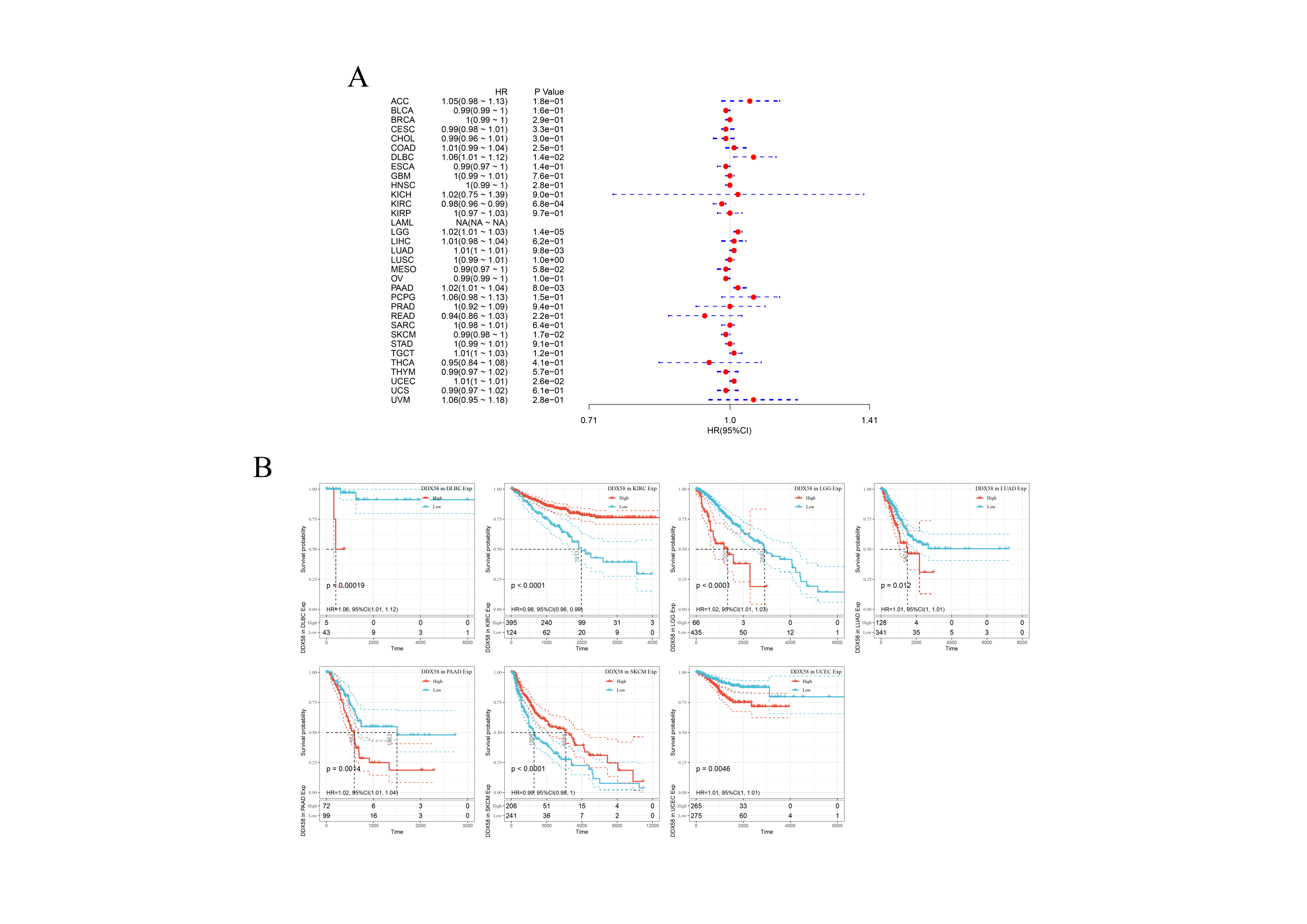

Supplement: Supplementary Figure 1 — Relationship between DDX58 expression levels and disease-specific survival (DSS) (A) Forest plot of the risk ratio of DDX58 in human pan-cancer; (B) Kaplan-Meier DSS curves of DDX58 in the seven most significantly associated tumors. [file Image_1.tif]

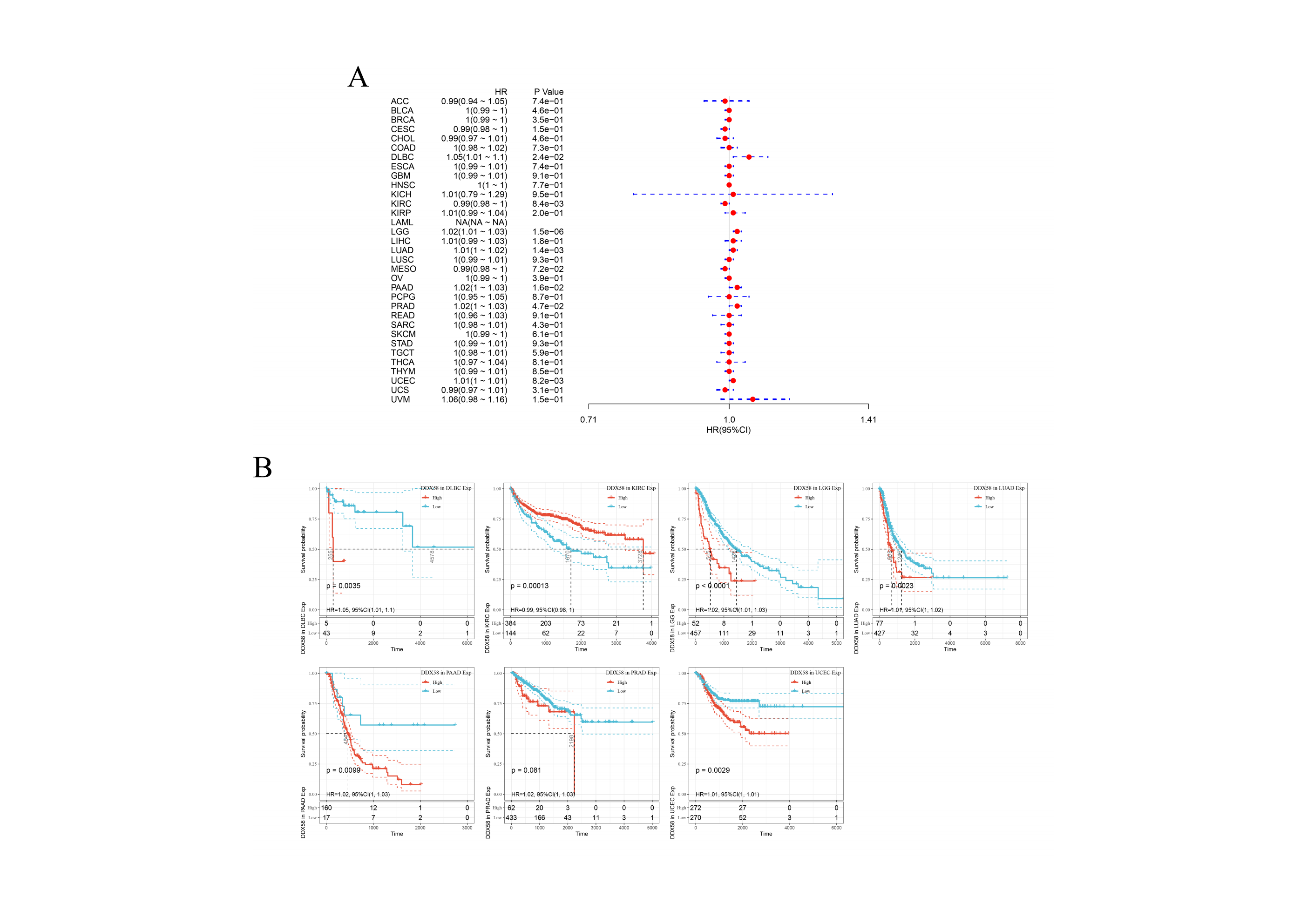

Supplement: Supplementary Figure 2 — Relationship between DDX58 expression levels and progression-free interval (PFI). (A) Forest plot of the risk ratio of DDX58 in human pan-cancer; (B) Kaplan-Meier PFI curves of DDX58 in the seven most significantly associated tumors. [file Image_2.tif]
